# The Mediating Roles of Upward Social Comparison and Self-esteem and the Moderating Role of Social Comparison Orientation in the Association between Social Networking Site Usage and Subjective Well-Being

**DOI:** 10.3389/fpsyg.2017.00771

**Published:** 2017-05-11

**Authors:** Jin-Liang Wang, Hai-Zhen Wang, James Gaskin, Skyler Hawk

**Affiliations:** ^1^Laboratory for Mental Health and Social Adaptation, School of Psychology, Southwest UniversityChongqing, China; ^2^Department of Tourism and Art for Humanity, Chongqing Youth and Vocational Technical CollegeChongqing, China; ^3^Marriott School of Management, Brigham Young University, ProvoUT, USA; ^4^Department of Educational Psychology, The Chinese University of Hong KongHong Kong, China

**Keywords:** social comparison orientation, upward social comparison, self-esteem, SNS usage, subjective well-being

## Abstract

The increased pervasiveness of social media use has raised questions about potential effects on users’ subjective well-being, with studies reaching contrasting conclusions. To reconcile these discrepancies and shed new light on this phenomenon, the current study examined: (1) whether upward social comparison and self-esteem mediate the association between social networking site (SNS) usage and users’ subjective well-being, and (2) whether the association between SNS usage and upward social comparison is moderated by users’ social comparison orientation. Data from 696 participants were collected. Structural equation modeling revealed that upward social comparison and self-esteem mediated the relationship between SNS usage and users’ subjective well-being. We found that social comparison orientation moderated the association between passive SNS usage and users’ upward social comparison. Specifically, social comparison orientation strengthened the association between passive SNS usage and upward social comparison. The results might suggest a process through which passive SNS usage is related to subjective well-being, and identify a context under which these associations may differ.

## Introduction

With the increasing popularity of social networking sites (SNSs), researchers have begun to investigate the relationship between SNS usage and users’ subjective well-being. However, mixed findings have been obtained. Some studies have demonstrated that non-excessive SNS usage can be positively related to users’ subjective well-being (e.g., Wang et al., 2014); whereas others have reported that SNS usage is associated with lower subjective well-being, such as depressive symptoms (Tandoc et al., 2015) and anxiety (Shaw et al., 2015). The factors behind the positive effects have received significant scholarly attention, and some studies have explored the negative effects (Krasnova et al., 2013, 2015; Appel et al., 2015). Despite this, further research is needed to understand under which circumstances SNS usage is related to lower subjective well-being. For example, moderators (such as social comparison orientation) and mediators (such as self-esteem and upward social comparison) might qualify the associations between SNS usage and psychological well-being (Lee, 2014; Chen et al., 2016). Social comparison can be upward or downward in nature. Upward comparison occurs when people compare themselves to someone they perceive to be superior (Wheeler, 1966), whereas a downward comparison is defined by making a comparison with someone perceived to be inferior (Wills, 1981). Buunk and Gibbons (2006) suggested that upward social comparisons naturally tend to induce more negative feelings.

The negative effects of SNS usage on individuals’ psychological well-being could be explained by the upward social comparisons that repeatedly occur on SNSs, which in turn decrease users’ self-esteem and then psychological well-being (Chen et al., 2016). As a platform offering abundant chances for impression management, such as the selection and editing of updates and photos, SNSs are filled with information exhibiting perfect happiness and flawless lives, which are actually exaggerated by users who upload this information (Walther, 2007). Individuals who view such information may feel personally inadequate (Jordan et al., 2011) and make poor self-evaluations (Chen et al., 2016). However, viewing this information might not necessarily always lead to negative feelings. Instead, this negative effect may depend on other possible mediating and moderating variables. For example, if SNS usage does not lead to upward social comparison, then the effect on self-esteem should be nominal, if at all present. Psychological traits (e.g., social comparison orientation) known to affect upward social comparison may have an impact on the association between SNS usage and upward social comparison. This implies that the relationship between SNS usage and subjective well-being may be more indirect than previously theorized. Therefore, in this study, we seek to clarify prior literature by theorizing and testing the process and context of this effect; namely, whether social comparison orientation moderates the association between passive SNS usage and upward social comparison, and whether upward social comparison mediates the relationship between passive SNS usage and users’ self-esteem, which is further related to subjective well-being.

## Theoretical Framework

### The Mediating Roles of Upward Social Comparison and Self-esteem on the Association between Passive SNS Usage and Subjective Well-Being

Researchers have suggested that humans are born with the tendency to compare themselves with others in order to assess their standing and their abilities (Festinger, 1954). The emergence of SNSs may reinforce this tendency, as they display an unprecedented volume of personal information that can be a powerful source of social comparison (Sabatini and Sarracino, 2015). As suggested by Sabatini and Sarracino (2015), social comparison relies on the availability of information about the lives of others. SNSs offer a fertile environment for upward social comparison, which in turn can lead to envy and negative feelings (e.g., Krasnova et al., 2015). The information presented on SNSs is more likely to lead to upward social comparison, since most of the information is idealized for the sake of impression management. The large amount of information presented on SNSs offers a platform for upward social comparison, and sometimes it is even hard for an individual to avoid evaluating themselves negatively compared to others (Lee, 2014; Tandoc et al., 2015). Therefore, it is possible that passively viewing information on SNS is related to more upward social comparison.

Since the idealistic and narcissistic information presented on SNSs has greatly enhanced social comparison norms, it is not surprising that users who spend more time on SNSs are more likely to agree that others have “better lives” and are “happier” than themselves (Chou and Edge, 2012). Researchers have also reported that individuals’ self-perceptions and self-evaluations tend to suffer after being exposed to profiles of attractive others on SNSs (Haferkamp and Krämer, 2011; Fardouly et al., 2015; Chen et al., 2016). Furthermore, recent studies have shown that individuals who passively use SNSs (e.g., mostly just viewing others profiles) were more likely to experience negative social comparison and negative affect (Chen et al., 2016), while active usage (e.g., posting regularly) was not related to negative social comparison or decreases in well-being (Appel et al., 2015; Tandoc et al., 2015). These findings suggest that differentiating between passive and active SNS usage is significant for understanding the influence of SNS usage on well-being (Verduyn et al., 2015).

The association between upward social comparison and users’ subjective well-being may not be direct, according to recent findings (Haferkamp and Krämer, 2011; Fardouly et al., 2015; Chen et al., 2016). The poor self-evaluations made after viewing others’ self-presentations on SNSs may lead them to feel less satisfied with their lives and to report more negative affect. Prior studies have shown that self-esteem is negatively related to depression and negative affect (Butler et al., 1994; Orth et al., 2008; Sowislo et al., 2014; Li et al., 2015), while positively related with life satisfaction (Diener and Diener, 1995) and positive affect (Watson et al., 2002; Orth et al., 2012). Therefore, self-esteem can predict users’ subjective well-being, and might be a mediating variable between upward social comparison and well-being.

Based on the aforementioned literature, we predict that passive SNS usage will be related to upward social comparison, and this comparison will further link to users’ self-esteem and then their subjective well-being. This leads to our first and second hypotheses:

H1. Upward social comparison mediates the relationship between the passive SNS usage and self-esteem.
H2. Self-esteem mediates the association between upward social comparison and subjective well-being.

### The Moderating Role of Social Comparison Orientation on the Association between Passive SNS Usage and Upward Social Comparison

As shown in prior studies, passive SNS usage does not necessarily lead to more frequent upward social comparison (de Vries and Kühne, 2015; Lup et al., 2015). One reason for these inconclusive findings might be that users’ social comparison orientation moderates the association between passive SNS usage and users’ upward social comparison. As suggested by Vogel et al. (2015), the effects of Facebook use differ based on social comparison orientation. Social comparison orientation refers to the extent to which individuals pay attention to, and base their own behavior on, the way others behave (Gibbons and Buunk, 1999). Several studies have reported a positive association between social comparison orientation and individuals’ social comparison frequency (e.g., Buunk et al., 2003, 2005). In one recent study, Lee (2014) found that individuals with high social comparison orientation are more likely to compare with others on SNSs than individuals with low social comparison orientation, perhaps because they recognize the abundant opportunities for social comparison available on social media (Vogel et al., 2015). In their experimental design, Vogel et al. (2015) found a moderating role of social comparison orientation when examining the impact of browsing the Facebook profile of an acquaintance on participants’ self-perceptions. Individuals with a high social comparison orientation are uncertain about themselves (Buunk and Gibbons, 2007) and might use the information available on Facebook for self-evaluation. Hence, they are more likely to compare with others after viewing SNS information. Therefore, we put forward our third hypothesis:

H3. Social comparison orientation moderates the association between passive SNS usage and upward social comparison.

Additionally, considering prior studies have reported gender and age differences in using SNSs (Muscanell and Guadagno, 2012; Thompson and Lougheed, 2012), we added gender and age as control variables in our model by regressing gender and age on passive SNS usage.

### SNSs in the Current Study

The type of SNS is an important factor to consider when examining the association between SNS usage and upward social comparison (de Vries and Kühne, 2015; Lup et al., 2015). For instance, de Vries and Kühne (2015) found that Facebook use was related to greater degrees of negative social comparison (i.e., upward social comparison), while Lup et al. (2015) reported that more frequent Instagram use was not associated with upward social comparison. The inconsistent findings might be explained by the different SNSs investigated and some other potential moderating variables. For example, Instagram, investigated in the study by Lup et al. (2015), provides different features from dominant SNSs like Facebook, such as allowing users to follow and/or be followed in a non-reciprocal way. This feature might contribute to the popularity of following celebrities with open profiles. Similarity to comparison standards and high personal relevance, both of which are more common among close friends and acquaintances, are two factors shown in prior research to promote social comparisons and envy (Smith and Kim, 2007). We chose to focus on Qzone and WeChat in the present study, because they focus on interaction with friends and acquaintances as opposed to following celebrities. Qzone and WeChat are the top two most popular sites among Chinese SNSs users. Qzone was created by Tencent in 2005. It allows users to write blogs, keep diaries, send photos, listen to music, and watch videos. WeChat is another SNS application developed by Tencent in 2011. As an instant messaging program, users can send texts, pictures, and audio and video. Users can additionally share their activities with their friends through the “Moments” feature, which is similar to posting information on one’s own Facebook profile. Most friends on WeChat lists are recommended from their mobile phone contacts and Qzone friends lists, therefore they are often acquaintances in the real world (as opposed to following just celebrities). In May 2015, it was estimated that around 549 million users logged in on WeChat every day (Curiosity China, 2015).

## Materials and Methods

### Participants and Procedures

In order to target Qzone and WeChat users, a hyperlink that directed to the survey website was posted in several local bulletin-board systems, online forums, and virtual communities. The survey targeted all possible SNSs user groups. Permission was obtained from the University’s Human Research Ethics Committee. Prior to answering the items, participants read information about the purpose of the study, implications of participation, and data protection. The information stressed that participation was completely voluntary and anonymous. Each participant received monetary compensation for participating (¥20, or about $3.00 USD). Of the 800 potential participants, 696 completed the survey (response rate = 87%), ranging in age from 17 to 24 years old, with an average age of 19.43 (*SD* = 1.65). Eight participants were under 18 years of age and permissions from their parents were obtained. A total of 132 (23.24%) participants identified themselves as male, 564 (76.76%) as female. The average time for completing the survey was 20 min.

### Measures

#### Passive SNS Usage

Passive SNS usage was measured by asking respondents three questions, which were “How frequently do you view others’ photos when logging on SNSs?,” “How frequently do you view others’ updates when logging on SNSs?,” and “How frequently do you view comments on your friends’ Wall when logging on SNSs?” Respondents rated each item on a 5-point Likert scale, ranging from 1 (*never*) to 5 (*almost every time I log on*).

#### Social Comparison Orientation

Social comparison orientation was measured by the social comparison orientation scale developed by Gibbons and Buunk (1999). This scale was developed to assess a person’s inclination to compare him/herself with others. The scale consists of eleven 5-point Likert scale items ranging from 1 (*strongly disagree*) to 5 (*strongly agree*). Example items include, “I often compare how my loved ones (boy or girlfriend, family members, etc.) are doing with how others are doing”; and “I always pay a lot of attention to how I do things compared with how others do things.”

#### Upward Social Comparison

Upward social comparison was captured by the negative upward social comparison affect scale developed by Buunk et al. (1990). Participants rated their feelings on the two items, which were “How often does it give you a pleasant feeling when you see that SNS friends live better lives than you do yourself? (reversed),” and “How often does it give you an unpleasant feeling when you see that SNS friends live better lives than you do yourself?” Both questions were rated on a 5-point scale ranging from 1 (*never*) to 5 (very *often*).

#### Self-esteem

Self-esteem was measured by a Chinese version of the 10-item Self-Esteem Scale by Rosenberg (1965), on a 5-point Likert scale ranging from 1 (*strongly disagree*) to 5 (*strongly agree*). Example items include “On the whole, I am satisfied with myself” and “I take a positive attitude toward myself.” Prior research has demonstrated good internal consistency, test–retest reliability, and sound validity (Blascovich and Tomaka, 1991).

#### Subjective Well-Being

Subjective well-being was computed by summing standardized scores of life satisfaction and positive affect, and then subtracting a standardized score of negative affect. Life satisfaction was assessed by the Satisfaction with Life Scale (SWLS; Diener et al., 1985). The SWLS includes five items such as, “In most ways, my life is close to my ideal” and “I am satisfied with life.” Participants were asked to indicate their agreement on a 5-point scale ranging from 1 (*strongly disagree*) to 5 (*strongly agree*). Positive affect and negative affect were measured by a Chinese version of the Positive and Negative Affect Scale (PNAS; Watson et al., 1988). Participants were asked to indicate the extent to which they had negative feelings or emotions in the past few days. Sample items include, “To what extent did you feel depressed in the past few days” and “To what extent did you feel active in the past few days,” ranging from 1 (*very slightly or not at all*) to 5 (*extremely*). This scale has shown good reliability and validity in prior research (Watson et al., 1988).

### Data Analytic Strategy

SPSS 21.0 and Mplus 7.0 were used for data analysis. Missing values were replaced by their means. Pearson correlation coefficients were used to assess the strengths of linear relationships between pairs of study variables. Before establishing the structural model, we conducted a confirmatory factor analysis to assess the goodness of fit of the measurement model with Mplus 7.0. The default estimation of maximum likelihood method was used. To study the adequacy of the estimated model, we used χ^2^/*df*, the root mean square error of approximation (RMSEA), the comparative fit index (CFI), the Tucker–Lewis coefficient (TLI), and the standardized root mean square residual (SRMR). χ^2^/*df* is greater than one and smaller than five as recommended by prior research (Salisbury et al., 2002). The CFI and TLI should be greater than 0.9 (Salisbury et al., 2002). For the RMSEA and SRMR, values less than 0.08 represent an acceptable fit (Byrne, 2006).

For the mediation model, we ran bootstrapping analysis to test for mediation, as simulation research found bootstrapping to be among the most powerful methods to detect mediation (Hayes, 2013). The process of bootstrapping creates a large sample from the original data (10,000 for this study) by a sampling with replacements strategy. It constructs a confidence interval (95% in this study) around the indirect effect, and the interval must not contain a zero to assume a significant indirect effect (Preacher and Hayes, 2008).

Regarding the moderation model, we calculated the continuous interaction term between passive SNS usage and social comparison orientation and included it in the model as a predictor of upward social comparison, and then used procedures recommended by Cohen et al. (2003) in interpreting the moderating effects between the two predictor variables on the dependent variable. We plotted the relationship between the independent variable (passive SNS usage) and the dependent variable (upward social comparison) when the levels of the moderator variable (social comparison orientation) were one standard deviation below and one standard deviation above mean value of the moderator variable. We also tested the statistical significance of each of these two slopes (Aiken et al., 1991), which represented the simple effect of the independent variable on the dependent variable at two levels of the moderator variable.

## Results

### Descriptive Statistics and Pearson Correlations

The descriptive statistics are presented in **Table 1**, along with the Pearson correlations between the variables of interest. Additionally, we conducted a *t*-test to compare the gender difference in passive SNS usage, and found that females scored significantly higher than males (*t* = 2.85, *p* < 0.01; *M*_male_= 3.24, *SD*_male_= 0.72, *M*_female_= 3.46, *SD*_female_= 0.69), although the actual difference in means was modest (only 0.22 on a 5-point scale). No significant gender difference was found on upward social comparison (*t* = 0.02, *p* > 0.05; *M*_male_ = 2.28, *SD*_male_ = 0.81, *M*_female_ = 2.28, *SD*_female_ = 0.66), self-esteem (*t* = 0.99, *p* > 0.05; *M*_male_ = 3.55, *SD*_male_ = 0.59, *M*_female_ = 3.61, *SD*_female_ = 0.60), social comparison orientation (*t* = 1.04, *p* > 0.05; *M*_male_ = 3.17, *SD*_male_ = 0.58, *M*_female_ = 3.22, *SD*_female_ = 0.54), and subjective well-being (*t* = 0.01, *p* > 0.05; *M*_male_ = 0.005, *SD*_male_ = 2.03, *M*_female_ = 0.003, *SD*_female_ = 2.06).

**Table 1:** Cronbach alphas reliabilities, means, standard deviations, and zero-order correlations of study variables (*N* = 696).

Variables	α	*M*	*SD*	2	3	4	5
(1) Passive SNS usage	0.70	2.32	0.54	0.13^∗∗^	0.10^∗^	-0.09^∗^	-0.01
(2) Social comparison orientation	0.75	3.21	0.55	1	0.14^∗∗^	-0.06	-0.07
(3) Upward social comparison	0.77	2.23	0.69	0.14^∗∗^	1	-0.15^∗∗^	-0.14^∗∗^
(4) Self-esteem	0.87	3.60	0.60	-0.06	-0.15^∗∗^	1	0.54^∗∗^
(5) Subjective well-being	0.74^a^, 0.86^b^, and 0.87^c^	0.00	2.05	-0.07	-0.14^∗∗^	0.54^∗∗^	1

*^∗^p < 0.05; ^∗∗^p < 0.01, ^a^refers to Satisfaction with Life Scale, ^b^refers to Positive Affect Subscale, and ^c^refers to Negative Affect Subscale.*

### Measurement Model

Result of the confirmatory factor analysis showed that the measurement model yielded an acceptable fit, with χ^2^/*df* = 2.88, *p* < 0.001, CFI = 0.91, TLI = 0.90, RMSEA = 0.05 (95% CI = [0.04, 0.05]), and SRMR = 0.06, after three items in social comparison orientation scale were deleted due to low factor loadings (below 0.30; i.e., “I am not the type of person who compares often with others,” “I often compare myself with others with respect to what I have accomplished in life,” and “I never consider my situation in life relative to that of other people”)^1^. Standardized factor loadings for the retained items ranged from 0.37 to 0.77 and were all significant at the *p* < 0.001 level. The reliability coefficient for the social comparison orientation scale was 0.80 after the items were deleted.

### Results Relating to Hypothesis 1 and Hypothesis 2: The Mediating Effects

**FIGURE 1:**
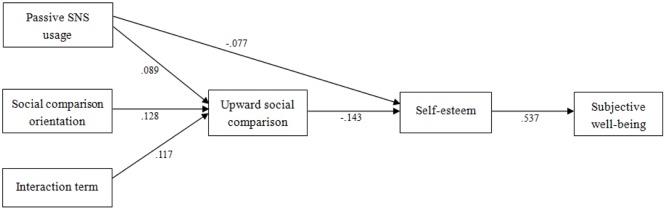
**Final structural model on the associations among passive social networking site (SNS) usage, social comparison orientation, upward social comparison, self-esteem, and subjective well-being.** This is a final structural model with amendments based on modification index, and non-significant paths were removed for the sake of clarity. All coefficients are standardized. Age and gender were excluded from the model due to non-significant coefficients. We also ran the analysis excluding the covariates and no significant changes were obtained.

The structural modeling was performed using Mplus 7.0. The model fit the data well (see **Figure 1** for details), with χ^2^/*df* = 1.38, *p* > 0.05, TLI = 0.98, CFI = 0.99, RMSEA = 0.02 (95% CI = [0.00, 0.05]), SRMR = 0.02. Passive SNS usage and social comparison orientation were positively related to upward social comparison (β = 0.09, *p* < 0.05 and β = 0.13, *p* < 0.001, respectively). We also added a direct path from passive SNS usage to subjective well-being in our model. The results showed that passive SNS usage was not directly related to subjective well-being, with β = 0.04, *p* > 0.05 (for the sake of clarity, this non-significant path was omitted in **Figure 1**).

The bootstrapping analysis found that the indirect effect of passive SNS usage on subjective well-being was significant (β = -0.05, *p* < 0.05), with 95% CI = -0.11 to -0.04. Passive SNS usage exerted indirect effects on subjective well-being through upward social comparison and self-esteem. Passive SNS usage predicted upward social comparison (β = 0.09, *p* < 0.01), which then predicted self-esteem (β = -0.14, *p* < 0.001), which then finally was associated with subjective well-being (β = 0.54, *p* < 0.001) (please see **Figure 1** for details). Upward social comparison was a significant mediator between passive SNS usage and self-esteem, 95% confidence interval (CI) = -0.026 to -0.006. Self-esteem also fully mediated the association between upward social comparison and subjective well-being, with 95% CI = -0.119 to -0.005. Additionally, according to the modification index, we added a link between passive SNS usage and self-esteem (β = -0.07, *p* < 0.05), considering that this is theoretically reasonable (for details, please see the “Discussion” section).

### Results Relating to Hypothesis 3: The Moderating Effect

**FIGURE 2:**
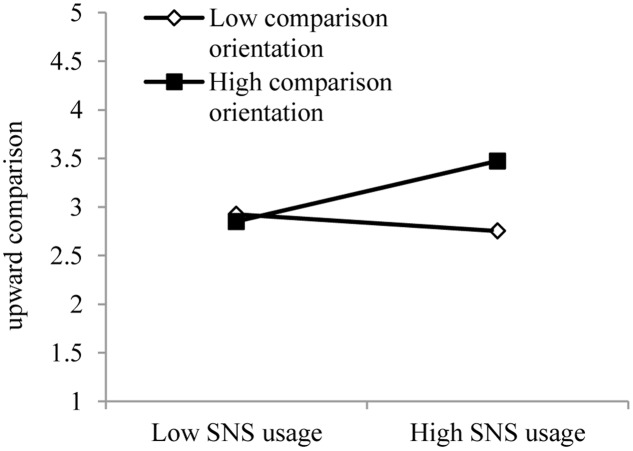
**The two-way interaction effect of passive SNS usage and upward social comparison**.

We found that the interaction term between passive SNS usage and social comparison orientation was positively related to upward social comparison (β = 0.12, *p* < 0.01). Based on the recommendations of Cohen et al. (2003) for interpreting the moderating effects between the two predictor variables on the dependent variable, we plotted the interaction between passive SNS usage and social comparison orientation (see **Figure 2**). We also calculated their corresponding simple effects using the PROCESS macro for SPSS by Hayes (2013). The results showed that for users with high social comparison orientation (Mean + 1SD), the association between passive SNS usage and upward social comparison was significant (β = 0.16, *p* < 0.05). However, for users with low social comparison orientation (Mean - 1SD), the association was not significant (β = 0.06, *p* > 0.05; see **Figure 2**).

## Discussion

In the present study, the results of structural modeling analysis suggested that passive SNS usage was positively related to upward social comparison, which in turn was associated with users’ lower self-evaluation and then their subjective well-being. These findings support Hypothesis 1 and 2, that upward social comparison and self-esteem are two mediators of the relationship between passive SNS usage and users’ subjective well-being. Additionally, we found that social comparison orientation moderated the association between passive SNS usage and upward social comparison, supporting Hypothesis 3. Social comparison orientation can strengthen the association between passive SNS usage and upward social comparison in SNS. These findings have improved our understanding of the context under which SNS usage can be associated with users’ subjective well-being, and can help to explain the mixed findings regarding the association between passive SNS usage and upward social comparison in extant literature (e.g., de Vries and Kühne, 2015; Lup et al., 2015). Our study suggests that following friends on SNS is related to more upward social comparison, while passively reviewing strangers’ information may not be (Lup et al., 2015). Our findings also provide implications for how interventions might diminish the negative effects for SNSs users, and the characteristics of users who are more susceptible to negative outcomes. Individuals with high social comparison orientation are more likely to make upward social comparisons, which may be related to poor self-evaluations and lower subjective well-being.

One goal of this study was to examine whether passive SNS usage is related to upward social comparison, and whether upward social comparison is related to lower self-esteem and subjective well-being. We found that passive SNS usage is positively associated with upward social comparison with SNS friends, which was further associated with more negative self-evaluations and more negative feelings. This is in line with previous studies (Vogel et al., 2014; Chen et al., 2016). For example, Vogel et al. (2014) reported that upward social comparison mediated the relationship between frequency of Facebook use and users’ self-esteem. In a recent study by Chen et al. (2016), self-esteem was found to mediate the negative association between passive SNS usage and users’ subjective well-being.

Upward social comparison mediated the relationships between passive SNS usage, on the one hand, and negative self-evaluation and lower subjective well-being on the other hand. This finding is reasonable, considering that SNS users are flooded with large amounts of exaggerated information exhibiting flawless lives by their SNS “friends,” which make upward social comparisons almost unavoidable (Appel et al., 2015; de Vries and Kühne, 2015). As suggested by prior studies, self-presentation on SNSs is often inspired by impression management (Walther, 2007) and the presented information is positively skewed (Jordan et al., 2011; Lee-Won et al., 2014). Therefore, using SNSs would likely increase the likelihood of unflattering social comparison (Appel et al., 2015; de Vries and Kühne, 2015). As a result, SNS users, especially passive SNS users, may experience upward social comparison, which is detrimental to perceptions about the self and self-esteem (Lee, 2014; de Vries and Kühne, 2015). The undermined self-esteem might be further related to lower life satisfaction and then associated with more negative feelings (Watson et al., 2002; Orth et al., 2012; Chen et al., 2016).

We also found a direct association between passive SNS usage and self-esteem. This link suggests that upward social comparison on SNSs is only one of the factors accounting for lower self-esteem, and that passive SNS usage can also directly predict undermined self-esteem. According to Leary et al. (1995), individuals establish their self-worth primarily from the feedback received from others. Self-esteem fluctuates in response to social feedback and social exclusion (Lamer et al., 2015), and can be shaped by social relationships (Leary et al., 1995). Users are less likely to gain feedback from, and communicate with, others when they engage in passive SNS activities, which may damage their self-esteem (Chen et al., 2016).

Our findings are in line with prior studies, which reported that SNS usage predicts decreased life satisfaction (Smith and Kim, 2007) and increased feelings of unfairness regarding the users’ own lives (Chou and Edge, 2012). However, our findings extend these prior studies by showing that passive SNSs use alone is not the reason for negative feelings. The reason for the effect is explained by the mediating roles of social comparison and suffered self-esteem. Our findings suggest that upward social comparison is positively related to the boastful information presented on SNSs, which is further associated with low life satisfaction and other negative feelings (e.g., envy and jealousy) among SNS users. Therefore, for people who experience negative feelings after using SNSs, eliminating upward social comparison could be an effective way to avoid this problem. For example, the “moments” feature on WeChat, through which self-presentation is posted, can be disabled. This could decrease users’ passive viewing.

Regarding the relationship between SNS usage and psychological well-being, prior studies have also suggested that SNS usage can improve users’ psychological well-being through the mediating effects of mood expression, social support, and self-disclosure (Special and Li-Barber, 2012; Liu and Yu, 2013; Wang et al., 2014). Our findings are not in conflict with these results when the complex mediating and moderating variables are considered. For example, the type of SNS usage may be an important moderator, as active SNS usage might be related to psychological well-being, while passive SNS usage might be linked to negative outcomes.

Our second goal was to examine whether social comparison orientation would moderate the relationship between passive SNS usage and upward social comparison. Our results indicate that individuals with high social comparison orientation are more likely to make upward social comparisons with others after viewing SNSs information. This is in line with a suggestion by Vogel et al. (2015) that the impact of SNS usage on users’ subjective well-being varies according to their social comparison orientation. Users with high social comparison orientation tend to pay more attention to others’ information and evaluate themselves based on others’ behavior, and therefore are more likely to compare themselves with others after viewing the abundant information on SNSs (Gibbons and Buunk, 1999). Another possible explanation for the close relationship between social comparison orientation and upward social comparison might be that individuals with high social comparison orientation tend to be uncertain about their self-worth, which provokes them to evaluate themselves by using information presented on SNSs. Moreover, Lee (2014) suggests that individuals with high social comparison orientation are more likely to make active upward social comparisons when exposed to social media, which also can explain the moderating role of social comparison orientation on the relationship between passive SNS usage and upward social comparison.

Our study is not without limitations. First, a cross-sectional design was used and, therefore, causality inferences cannot be made. These questions of causality could be further informed by a longitudinal design, or supported directly through experimentation. Additionally, the sample size of 696 is hardly representative of the full population of SNSs users (over a billion worldwide). However, the sample we obtained is somewhat indicative of the “typical” young SNS user. As such, while caution should be exercised when trying to broadly apply the findings from this study, we might say the results are perhaps informative regarding this type of population. Moreover, further studies should consider other factors (e.g., social anxiety) relevant to passive vs. active SNS usage in their models to explain the relationship between SNS usage and subjective well-being, given the small effects obtained in the current study.

Despite its limitations, our study has investigated the mechanisms by which SNS usage is negatively related to users’ subjective well-being, by adding social comparison orientation, upward social comparison, and self-esteem into our model. We found that upward social comparison is an important variable associated with negative outcomes among individuals after using SNSs. These results highlight the need to consider decreasing unflattering social comparison as a component of interventions for users suffering from lower subjective well-being. Our findings also imply that high social comparison orientation SNSs users are more likely to make more upward social comparisons, and therefore suffer regarding their self-evaluations and subjective well-being. Hence, high social comparison orientation individuals should especially consider decreasing passive SNS usage.

## Ethics Statement

This study was carried out in accordance with the recommendation of the University’s Human Research Ethics Committee with written informed consent from all subjects. All subjects gave written informed consent in accordance with the Declaration of Helsinki. The protocol was approved by the University’s Human Research Ethics Committee.

## Author Contributions

J-LW proposed the research idea and designed the study, as well as drafted the manuscript; H-ZW collected the data and revised it; JG analyzed and interpreted the data; SH revised the article critically.

## Conflict of Interest Statement

The authors declare that the research was conducted in the absence of any commercial or financial relationships that could be construed as a potential conflict of interest.

## Footnotes

1 We also ran a moderation analysis that included the three items of social comparison orientation and no significant discrepancies were found between the two sets of results.
